# Influence of early myofunctional training on maxillary arch development in children aged 4–7 years with mild oral habits: a controlled pilot study

**DOI:** 10.3389/fped.2026.1809806

**Published:** 2026-07-07

**Authors:** Qiwen Xiao, Junqing Ma

**Affiliations:** 1 Orthodontics Department, Nanjing Medical University Affiliated Stomatological Hospital, Nanjing City, Jiangsu Province, China; 2 State Key Laboratory Cultivation Base of Research, Prevention and Treatment for Oral Diseases, Nanjing City, Jiangsu Province, China; 3 Jiangsu Province Engineering Research Center of Stomatological Translational Medicine, Nanjing City, Jiangsu Province, China

**Keywords:** children, maxillary arch development, myofunctional therapy, oral habits, orofacial myology, preventive orthodontics

## Abstract

**Background:**

Thumb sucking, the use of pacifier and mouth breathing in the early childhood may negatively influence the development of the maxillary arch. Myofunctional therapy has become a non-invasive intervention that can fulfil the dysfunction of the orofacial muscles, although the studies on its effectiveness in preventing arch underdevelopment are limited.

**Methods:**

The sample size (*n* = 64) was divided into two groups: the intervention group (*n* = 32) where the children were subjected to 12 months of structured myofunctional training and habit cessation counselling, and the control group (*n* = 32) where the children were subjected to habit cessation counselling only. Baseline, 6 months, and 12 months were made of dental impression and digital measurements of maxillary intercanine width, intermolar width and arch perimeter and palatal depth. Validated questionnaires and clinical examination protocol were used to measure oral habit frequency and orofacial muscle functioning.

**Results:**

After 12 months, the intervention group showed significantly greater increases in intercanine width (3.42 ± 1.18 mm vs. 1.87 ± 0.95 mm; *p* < 0.001) and intermolar width (2.76 ± 1.04 mm vs. 1.45 ± 0.88 mm; *p* < 0.001) compared with controls. Arch perimeter increased by 4.28 ± 1.56 mm in the intervention group vs. 2.34 ± 1.21 mm in controls (*p* < 0.001). Habit cessation was also higher in the intervention group (87.5% vs. 65.6%; *p* = 0.038). Orofacial muscle function scores improved significantly more in the intervention group (*p* < 0.001).

**Conclusion:**

These findings provide preliminary evidence that early myofunctional training may have a beneficial effect on maxillary arch development in children with mild oral habits and may support preventive orthodontic strategies.

## 1 Introduction

Craniofacial complex in early childhood is developed under a complex of genetic, environmental, and functional factors (1). Among these, oral habits and orofacial muscle activity are very important in determining the dental arches and development of the correct occlusion (2). Unhealthy oral habits such as non-nutritive sucking, tongue thrusting, and breathing with the mouth have always been linked with changes in the structure of the maxillary arch, such as palate narrowing, overjet, and anterior open bite formation (3).

The maxillary arch grows and develops greatly in first decade of life, and transverse dimension is highly vulnerable to environmental factors prior to the onset of the ossification of the transverse suture at the midpalatal position (4). Studies indicate that persistent oral habits after the age of three years can exert sufficient force to alter normal maxillary growth, potentially leading to malocclusion and functional impairment (5). It is confirmed that oral habits among the preschool and early school-age children are prevalent with the ranges ranging between 25% and 60% in various groups pointing to the importance of the topic of oral habits to the population health (6).

Myofunctional therapy, also known as orofacial myofunctional therapy (OMT), is a set of exercises and other methods that help to encourage improved muscle functioning patterns in the area of the face and mouth (7). The objectives of this form of therapy are to correct good tongue position, nasal breathing, lips seal abilities, and well-coordinated swallowing patterns (8). The main idea of myofunctional therapy is founded on the theory of functional matrix, which was introduced by Moss and which suggests that the skeletal growth and development are defined largely by the functionality of soft tissues (9).

The recent studies have outlined the possible involvement of myofunctional therapy in the treatment of diverse orofacial dysfunctions. Researches have proven the acceptance in the symptoms of obstructive sleep apnea (10), temporomandibular disorders (11), and speech articulation problems (12) after the myofunctional intervention. Also, studies have proposed myofunctional exercises to be used in conjunction with orthodontic care which can possibly affect the period of treatment and maintain stability of results (13).

It has been widely researched on the relationship between the orofacial muscle function and dental arch development. Proffit and colleagues presented the equilibrium theory, stating that the relative position that forces of tongue, lips and cheeks provide dictate the position of teeth and arch configuration (14). When this balance is destroyed due to the abnormal functioning of the muscles or the habits, dental and skeletal changes can follow. Research that has studied the mouth breathing of children has always reported a smaller maxillary arch and elevated palatal vaults when compared to the nasal breathing controls (15).

Although there is an increasing interest in preventive and interceptive orthodontic interventions, specific controlled clinical studies on the effect of myofunctional training on arch development in young children with oral habits are sparse (16). In the literature, most studies have been done on older children or those who already have malocclusion, and preventive intervention has not addressed the most suitable stage of growth when prevention can be most effective (17). Also, most of the studies that have been conducted in the past have been short-limited due to small sample sizes, absence of control groups or inadequate follow-up time (18).

The period during which the maxillary transverse develops best is at the early stages of a child and thus it is possible that the best results of intervention during this period may produce the best outcome (19). Oral habits can be identified and controlled early on with myofunctional training to achieve a good orofacial muscle functioning, which is associated with preventing or limiting the development of abnormalities of dental arch (20). Nevertheless, the precise influences of structured programs of myofunctional training on objective parameters of maxillary arch development have not been properly described in controlled research.

Moreover, the counselling no habit cessation as a single intervention has demonstrated inconsistent success, but the implementation of myofunctional exercises could improve the success rate of the therapy since underlying muscle dysfunction could be the cause of the habit or habit effects (21). A clear insight into the existence of any quantifiable advantages of early myofunctional intervention over the typical counselling strategies is necessary in order to design evidence-based clinical procedures.

This controlled pilot study was designed to determine the effects of an organized early myofunctional training intervention on the development of the maxillary arch of children with mild oral habits aged 4–7 years. In particular, this research aimed at comparing the variations in maxillary intercanine width, intermolar width, arch perimeter, and palatal depth in children who received myofunctional training with habit cessation counselling compared to those who were only provided with habit cessation counselling in 12 months. Secondary outcomes were the evaluation of cessation rates of habits and improvement of the orofacial muscle functioning between the groups.

## 2 Materials and methods

### 2.1 Study design

This prospective controlled pilot study was conducted at a paediatric dental clinic affiliated with a university teaching hospital between January 2022 and June 2023. The study protocol was reviewed and approved by the Institutional Ethics Committee (Protocol Reference: SU202112067) and the research was conducted in accordance with the principles outlined in the Declaration of Helsinki. Written informed consent was obtained from the parents or legal guardians of all participating children, and verbal assent was obtained from children aged 6 years and older.

### 2.2 Sample size determination

Sample size calculation was performed based on preliminary data from a pilot sample and previous literature on maxillary arch dimensional changes. Assuming a clinically significant difference of 1.5 mm in intercanine width change between groups, a standard deviation of 1.8 mm, an alpha level of 0.05, and a power of 80%, a minimum sample size of 28 participants per group was calculated. To account for potential dropouts and incomplete data, 32 participants were enrolled in each group, yielding a total sample of 64 children.

### 2.3 Participant selection

Children were recruited from routine paediatric dental examinations and referrals from general paediatric practices. Potential participants were screened using a structured interview with parents and clinical examination by a calibrated paediatric dentist.

Children were classified as having mild oral habits when the habit frequency was intermittent or part-time in nature, typically reported as “sometimes” or “often,” but not “always,” in the parental questionnaire. The daily duration of the habit was less than 6 h per day, and the behavior did not occur continuously during sleep. In addition, children showed no evidence of established severe malocclusion, defined as overjet ≤6 mm, anterior open bite ≤3 mm, and absence of posterior crossbite. Participants also demonstrated no clinically significant skeletal maxillary constriction and no functional impairment requiring immediate orthodontic intervention. Habit identification was based on parental interview, clinical examination, and a validated habit frequency questionnaire. Children exhibiting continuous habits, prolonged nighttime habits, or associated structural malocclusion were classified as having moderate-to-severe habits and were excluded from the study.

**Inclusion Criteria:**

- Age between 4 years 0 months and 7 years 11 months at enrolment
- Presence of at least one mild oral habit (thumb/finger sucking, prolonged pacifier use, lip sucking/biting, or habitual mouth breathing) for a minimum of 6 months
- Primary or early mixed dentition stage
- Generally healthy without significant systemic conditions
- Parents/guardians willing to participate in the training program and attend scheduled follow-up appointments
- No previous orthodontic or myofunctional therapy

**Exclusion Criteria:**

- Severe oral habits with already established significant malocclusion (overjet >6 mm, anterior open bite >3 mm, or posterior crossbite)
- Craniofacial syndromes or anomalies
- Diagnosed neuromuscular disorders
- Significant adenotonsillar hypertrophy requiring surgical intervention
- Developmental delays affecting ability to perform exercises
- Current speech therapy or other orofacial interventions
- Allergies to impression materials

### 2.4 Group allocation

Participants were allocated to either the intervention or control group using a quasi-randomized method based on the week of enrolment (odd weeks: intervention group; even weeks: control group). The allocation schedule was predefined before recruitment and applied consecutively to all eligible participants. Eligibility screening and consent procedures were completed prior to group assignment to minimize allocation bias. The recruiting clinician followed the fixed schedule without discretion to alter assignments. Although formal allocation concealment was not implemented, baseline demographic and clinical characteristics were compared between groups to assess allocation comparability.

### 2.5 Intervention protocol

#### 2.5.1 Intervention group

Children in the intervention group received a comprehensive 12-month myofunctional training program in addition to standard habit cessation counseling. The protocol was adapted from established orofacial myofunctional therapy principles for young children and progressed in three phases. All exercises were demonstrated in-office, supervised by a certified orofacial myofunctional therapist, and reinforced with parent education, visual aids, and gamification (e.g., sticker charts, animal-themed cues) to enhance engagement in this age group.

- **Initial Phase (Weeks 1–4):** Two in-office sessions focused on foundational awareness and basic muscle activation. Key exercises included:
  - Lip strengthening: Button pull exercise (holding a small, age-appropriate button or soft toy attached to a string with the lips while breathing nasally; 5–10 repetitions, 5–10 s hold).
  - Lip stretching and seal: Gentle lip closure without mentalis strain, progressing to holding a tongue depressor or similar between the lips.
  - Tongue posture awareness: “Tongue spot” placement (resting the tongue tip on the incisive papilla behind the upper incisors with lips closed and nasal breathing; hold 10 s, 10 repetitions).
  - Basic nasal breathing exercises: Encouraging nose breathing during short activities with mouth closed. Daily home practice: 10–15 min, twice daily, under parental supervision.
- **Active Training Phase (Months 2–6):** Monthly in-office sessions for progression and compliance checks. Exercises were advanced to build strength, coordination, and functional integration:
  - Tongue elevation and lateralization: Tongue push-ups against the palate or “painting” the roof of the mouth with the tongue tip (10–15 repetitions).
  - Cheek resistance: Puffing cheeks or holding air while maintaining lip seal.
  - Swallowing pattern training: Correct swallow with tongue on spot, teeth lightly together, and lips sealed (no tongue thrust); practiced with small dissolvable items or during meals.
  - Breathing exercises: Prolonged nasal breathing with lip seal during play or reading. Daily home program: 10–15 min, twice daily, with progressive difficulty.
- **Maintenance Phase (Months 7–12):** Bi-monthly in-office visits to reinforce habits and reduce supervision. Exercises focused on integration into daily routines with lower frequency (10 min once daily). Emphasis was placed on automatic proper tongue rest posture, competent lip seal, and nasal breathing during rest and activity.

### 2.6 Outcome measures and data collection

#### 2.6.1 Primary outcomes—maxillary arch measurements

Dental impressions were obtained using alginate impression material (Tropicalgin, Zhermack) in appropriately sized pediatric trays at baseline (T0), 6 months (T1), and 12 months (T2). Impressions were poured immediately with Type IV dental stone, and models were trimmed according to standard protocol.

All measurements were performed on digital scans of dental models using a laboratory scanner (3Shape D700) and measurement software (3Shape OrthoAnalyzer). The following parameters were assessed:

1. **Intercanine Width (ICW):** Distance between the cusp tips of the primary maxillary canines
2. **Intermolar Width (IMW):** Distance between the mesiopalatal cusp tips of the maxillary second primary molars or first permanent molars if erupted
3. **Arch Perimeter (AP):** Length of a smooth curve passing through the contact points from the distal surface of the second primary molar on one side to the corresponding point on the opposite side
4. **Palatal Depth (PD):** Perpendicular distance from a line connecting the palatal gingival margins of the second primary molars to the deepest point of the palatal vault

**Secondary Outcomes:**

1. **Habit Frequency Assessment:** Parents completed a validated habit frequency questionnaire at each time point, recording the frequency (never, rarely, sometimes, often, always) and duration of observed habit behaviours.
2. **Orofacial Muscle Function Evaluation:** Clinical assessment of orofacial muscle function was performed using a modified Nordic Orofacial Test-Screening (NOT-S) protocol adapted for children. Assessment included evaluation of: lip seal competence, tongue posture and mobility, breathing pattern, swallowing pattern, and facial muscle symmetry. A composite score ranging from 0 (severe dysfunction) to 12 (optimal function) was assigned.
3. **Compliance Assessment:** For the intervention group, compliance with the exercise program was monitored using parent-completed exercise logs and therapist observations. Compliance was categorized as high (>80% of prescribed exercises), moderate (50%–80%), or low (<50%).

Adherence to the intervention and habit frequency were primarily assessed through structured parental reporting forms completed at each follow-up visit. Parents were instructed to record daily appliance use and habit occurrence using standardized checklists to minimize recall bias. At each visit, the investigator reviewed the completed forms with caregivers to verify entries and resolve inconsistencies. In addition, clinical indicators such as appliance wear patterns and intraoral findings were used as supportive measures to qualitatively corroborate reported compliance. Although these procedures were implemented to improve reporting accuracy, parental reporting remained the primary source for some secondary outcomes.

Breathing pattern was clinically classified at each assessment time point as nasal, mixed, or mouth breathing using a standardized protocol adapted from pediatric orofacial myofunctional evaluation guidelines. Assessments were conducted by a calibrated examiner while the child was at rest (seated and standing positions) and during quiet activities. Key observational criteria included:

- **Nasal breathing**: Competent lip seal (lips gently closed without strain or mentalis muscle activity), closed-mouth posture, and predominant nasal airflow with no audible or visible oral breathing.
- **Mouth breathing**: Habitual or predominant oral airflow, open-mouth posture at rest, incompetent lip seal, and visible or audible mouth breathing.
- **Mixed breathing**: Alternating or combined nasal and oral airflow, occasional open-mouth posture with partial nasal breathing, or inconsistent patterns during observation.

These observations were corroborated by parental responses on the validated habit frequency questionnaire regarding daytime and nighttime breathing behaviors (e.g., sleeping with mouth open, dry mouth upon awakening, or frequent open-mouth posture during play). The classification aligns with common clinical practices in orofacial myology, emphasizing habitual posture and functional breathing mode rather than momentary forced breathing. All assessments were performed to the extent possible by an examiner blinded to group allocation.

### 2.7 Examiner calibration and blinding

All dental arch measurements were performed by a single trained examiner who was blinded to group allocation. Examiner calibration was performed on 10 models not included in the study, with repeat measurements taken one week apart. Intra-examiner reliability was assessed using intraclass correlation coefficients (ICC), with all values exceeding 0.90, indicating excellent reliability.

Clinical assessments of orofacial muscle function were performed by a calibrated examiner different from the myofunctional therapist providing the intervention. Complete blinding was not possible due to the nature of the intervention, but outcome assessors were kept unaware of group assignment to the extent feasible.

### 2.8 Statistical analysis

All statistical analyses were performed using SPSS software version 26.0 (IBM Corporation, Armonk, NY, USA). Descriptive statistics were calculated for all variables, with continuous data expressed as mean ± standard deviation (SD) and categorical data as frequencies and percentages. Normality of continuous variables (e.g., arch dimensions and changes) was assessed at each time point using the Shapiro–Wilk test, which is particularly suitable for small sample sizes (*n* = 32 per group). Homogeneity of variances was evaluated with Levene's test. Baseline comparability between groups was evaluated using independent samples *t*-tests for continuous variables and chi-square tests for categorical variables. Changes in arch dimensions (intercanine width, intermolar width, arch perimeter, and palatal depth) from baseline to each follow-up time point were calculated for each participant. Between-group comparisons of these dimensional changes were performed using independent samples *t*-tests, as the data met normality assumptions. Within-group changes over time (baseline, 6 months, 12 months) were assessed using repeated measures analysis of variance (ANOVA) with Bonferroni *post-hoc* corrections for multiple comparisons. Sphericity was checked using Mauchly's test, and the Greenhouse-Geisser correction was applied if violated. For orofacial muscle function scores (ordinal data from the modified NOT-S protocol), the Mann–Whitney *U* test was used for between-group comparisons, and the Wilcoxon signed-rank test was applied for within-group changes, consistent with the non-parametric nature of the data. Habit cessation rates (categorical) were compared using chi-square tests or Fisher's exact test when expected cell counts were low. Multiple linear regression analysis was performed to identify factors associated with change in intercanine width (primary outcome), including age, gender, type and duration of habit, baseline measurements, and group allocation as independent variables. Effect sizes were calculated using Cohen's *d* for between-group comparisons of continuous outcomes (interpreted as small: 0.2, medium: 0.5, large: 0.8). Missing data due to loss to follow-up were handled using an intention-to-treat approach with last observation carried forward. A two-tailed significance level was set at *p* < 0.05 for all analyses.

## 3 Results

### 3.1 Participant characteristics and flow

Of the 72 children initially screened, 64 met the inclusion criteria and were enrolled in the study (32 per group). During the 12-month study period, 3 participants in the intervention group and 2 participants in the control group were lost to follow-up, resulting in completion rates of 90.6% and 93.8%, respectively. All available data were included in the analysis using an intention-to-treat approach with last observation carried forward for missing values.

Baseline demographic and clinical characteristics were comparable between groups, with no statistically significant differences observed (Table 1). The mean age was 5.43 ± 1.12 years in the intervention group and 5.51 ± 1.08 years in the control group. The distribution of oral habit types was similar between groups, with thumb/finger sucking being the most common habit (43.8% and 40.6% in intervention and control groups, respectively).

**Table 1:** Baseline characteristics of study participants.

Variable	Intervention group (*n* = 32)	Control group (*n* = 32)	*p*-value
Age (years), mean ± SD	5.43 ± 1.12	5.51 ± 1.08	0.776
Gender, *n* (%)			0.612
Male	17 (53.1)	15 (46.9)	
Female	15 (46.9)	17 (53.1)	
Type of Oral Habit, *n* (%)			0.894
Thumb/finger sucking	14 (43.8)	13 (40.6)	
Prolonged pacifier use	8 (25.0)	9 (28.1)	
Lip sucking/biting	5 (15.6)	6 (18.8)	
Mouth breathing	5 (15.6)	4 (12.5)	
Duration of Habit (years), mean ± SD	3.21 ± 1.45	3.08 ± 1.38	0.716
Baseline ICW (mm), mean ± SD	26.84 ± 2.15	27.02 ± 2.08	0.738
Baseline IMW (mm), mean ± SD	42.56 ± 2.67	42.78 ± 2.54	0.742
Baseline AP (mm), mean ± SD	68.42 ± 4.23	68.87 ± 4.18	0.673
Baseline PD (mm), mean ± SD	14.82 ± 1.56	14.68 ± 1.48	0.715
Baseline NOT-S Score, mean ± SD	7.28 ± 1.82	7.41 ± 1.76	0.772

ICW, intercanine width; IMW, intermolar width; AP, arch perimeter; PD, palatal depth; NOT-S, nordic orofacial test-screening.

Baseline intercanine relationships and anteroposterior occlusal features were also comparable between groups. Mean overjet was 2.8 ± 1.1 mm in the intervention group and 2.9 ± 1.2 mm in the control group (*p* = 0.812). No participant in either group exhibited significant Class II malocclusion (overjet >6 mm or full-cusp Class II molar relationship), consistent with the study's focus on mild oral habits without established severe malocclusion. Minor anteroposterior variations (e.g., edge-to-edge or mild end-on relationships) occurred at similar low frequencies in both groups and showed no statistically significant difference (*p* > 0.05). These findings confirm baseline equivalence in the transverse and anteroposterior dimensions assessed from dental models and clinical examination.

### 3.2 Changes in maxillary arch dimensions

Significant changes in maxillary arch dimensions were observed in both groups over the 12-month study period; however, the magnitude of change was significantly greater in the intervention group for all primary outcome measures (Table 2).

**Table 2:** Changes in maxillary arch dimensions over 12 months.

Parameter	Time point	Intervention group (*n* = 32) Mean ± SD	Control group (*n* = 32) Mean ± SD	Difference (95% CI)	*p*-value
Intercanine Width (mm)					
	Baseline	26.84 ± 2.15	27.02 ± 2.08	−0.18 (−1.23, 0.87)	0.738
	6 months	29.02 ± 2.24	28.14 ± 2.12	0.88 (−0.21, 1.97)	0.112
	12 months	30.26 ± 2.31	28.89 ± 2.18	1.37 (0.25, 2.49)	0.017
	Change (T0-T2)	3.42 ± 1.18	1.87 ± 0.95	1.55 (1.02, 2.08)	<0.001
Intermolar Width (mm)					
	Baseline	42.56 ± 2.67	42.78 ± 2.54	−0.22 (−1.52, 1.08)	0.742
	6 months	44.12 ± 2.71	43.56 ± 2.58	0.56 (−0.76, 1.88)	0.398
	12 months	45.32 ± 2.78	44.23 ± 2.62	1.09 (−0.26, 2.44)	0.112
	Change (T0-T2)	2.76 ± 1.04	1.45 ± 0.88	1.31 (0.83, 1.79)	<0.001
Arch Perimeter (mm)					
	Baseline	68.42 ± 4.23	68.87 ± 4.18	−0.45 (−2.55, 1.65)	0.673
	6 months	70.86 ± 4.31	70.24 ± 4.22	0.62 (−1.51, 2.75)	0.564
	12 months	72.70 ± 4.42	71.21 ± 4.28	1.49 (−0.69, 3.67)	0.178
	Change (T0-T2)	4.28 ± 1.56	2.34 ± 1.21	1.94 (1.25, 2.63)	<0.001
Palatal Depth (mm)					
	Baseline	14.82 ± 1.56	14.68 ± 1.48	0.14 (−0.62, 0.90)	0.715
	6 months	14.58 ± 1.52	14.72 ± 1.46	−0.14 (−0.88, 0.60)	0.708
	12 months	14.34 ± 1.48	14.80 ± 1.44	−0.46 (−1.19, 0.27)	0.214
	Change (T0-T2)	−0.48 ± 0.62	0.12 ± 0.54	−0.60 (−0.89, −0.31)	<0.001

T0 = baseline; T2 = 12 months. Positive changes indicate increases; negative values for palatal depth indicate reduction (flattening). Cohen's *d* effect sizes for T0–T2 changes: intercanine width = 1.45; intermolar width = 1.36; arch perimeter = 1.39; palatal depth = 1.02 (all large effects).

#### 3.2.1 Intercanine width

The intervention group showed a mean increase of 3.42 ± 1.18 mm from baseline to 12 months, compared to 1.87 ± 0.95 mm in the control group. This difference was statistically significant (*p* < 0.001, Cohen's *d* = 1.45) (Table 3). At 6 months, the difference was already apparent (2.18 ± 0.89 mm vs. 1.12 ± 0.72 mm; *p* < 0.001).

**Table 3:** Mean changes in maxillary arch dimensions from baseline to 12 months (T0–T2).

Parameter	Intervention group mean change ± SD (mm)	Control group mean change ± SD (mm)	Between-group difference (95% CI)	*p*-value	Cohen's *d*
Intercanine Width	3.42 ± 1.18	1.87 ± 0.95	1.55 (1.02, 2.08)	<0.001	1.45
Intermolar Width	2.76 ± 1.04	1.45 ± 0.88	1.31 (0.83, 1.79)	<0.001	1.36
Arch Perimeter	4.28 ± 1.56	2.34 ± 1.21	1.94 (1.25, 2.63)	<0.001	1.39
Palatal Depth	−0.48 ± 0.62	0.12 ± 0.54	−0.60 (−0.89, −0.31)	<0.001	1.02

#### 3.2.2 Intermolar width

The intervention group demonstrated a mean increase of 2.76 ± 1.04 mm compared to 1.45 ± 0.88 mm in controls (*p* < 0.001, Cohen's *d* = 1.36). Progressive increases were observed at both time points in the intervention group.

#### 3.2.3 Arch perimeter

Arch perimeter increased by 4.28 ± 1.56 mm in the intervention group vs. 2.34 ± 1.21 mm in the control group over 12 months (*p* < 0.001, Cohen's *d* = 1.39).

#### 3.2.4 Palatal depth

Changes in palatal depth showed a different pattern, with the intervention group showing a slight decrease (−0.48 ± 0.62 mm), indicating a less vaulted palate, while the control group showed minimal change (0.12 ± 0.54 mm). This difference was statistically significant (*p* < 0.001).

Longitudinal changes in maxillary arch dimensions over the 12-month study period are illustrated in Supplementary Figure S1. Progressive increases in intercanine width, intermolar width, and arch perimeter were observed in both groups; however, the magnitude of change was consistently greater in the intervention group. Divergence between groups became evident at 6 months and further increased at 12 months. In contrast, palatal depth demonstrated a reduction in the intervention group, whereas minimal change was observed in controls. As shown in Supplementary Figure S1, the intervention group exhibited greater transverse expansion and arch perimeter increase across all time points compared with the control group, with clear separation of trajectories by 6 months.

### 3.3 Secondary outcomes

#### 3.3.1 Habit cessation

Complete cessation of the primary oral habit was achieved by 28 children (87.5%) in the intervention group compared to 21 children (65.6%) in the control group by 12 months. This difference was statistically significant (*χ*^2^ = 4.31, *p* = 0.038). Partial reduction in habit frequency was observed in 3 additional children in each group (Table 4).

**Table 4:** Secondary outcome measures at baseline and 12 months.

Variable	Intervention group (*n* = 32)	Control group (*n* = 32)	*p*-value
Habit cessation at 12 months, *n* (%)			
Complete cessation	28 (87.5)	21 (65.6)	0.038*
Partial reduction	3 (9.4)	6 (18.8)	
No change	1 (3.1)	5 (15.6)	
NOT-S score, mean ± SD			
Baseline	7.28 ± 1.82	7.41 ± 1.76	0.772
6 months	9.12 ± 1.45	7.89 ± 1.62	0.002
12 months	10.56 ± 1.24	8.45 ± 1.52	<0.001
Change (T0-T2)	3.28 ± 1.36	1.04 ± 0.92	<0.001
Breathing pattern at 12 months, *n* (%)			
Nasal breathing	29 (90.6)	23 (71.9)	0.052
Mixed breathing	3 (9.4)	7 (21.9)	
Mouth breathing	0 (0.0)	2 (6.3)	
Lip seal competence at 12 months, *n* (%)			
Competent	30 (93.8)	24 (75.0)	0.039*
Incompetent	2 (6.3)	8 (25.0)	
Compliance (intervention group only), *n* (%)			
High (>80%)	22 (68.8)	—	—
Moderate (50%–80%)	7 (21.9)	—	—
Low (<50%)	3 (9.4)	—	—

NOT-S, nordic orofacial test-screening; T0, baseline; T2, 12 months Chi-square test for categorical variables; Mann–Whitney *U* test for ordinal variables.

#### 3.3.2 Orofacial muscle function

Significant improvements in orofacial muscle function scores were observed in the intervention group over the study period. The NOT-S score increased from 7.28 ± 1.82 at baseline to 10.56 ± 1.24 at 12 months in the intervention group, compared to an increase from 7.41 ± 1.76 to 8.45 ± 1.52 in the control group. The between-group difference at 12 months was statistically significant (*p* < 0.001).

#### 3.3.3 Compliance

Among participants in the intervention group, compliance with the exercise program was categorized as high in 22 children (68.8%), moderate in 7 children (21.9%), and low in 3 children (9.4%). Higher compliance was associated with greater improvements in arch dimensions, though this relationship did not reach statistical significance in regression analysis.

The overall magnitude of change from baseline to 12 months is summarized in Supplementary Figure S2. The intervention group demonstrated significantly greater increases in intercanine width, intermolar width, and arch perimeter compared with controls. Additionally, palatal depth decreased in the intervention group, whereas a slight increase was observed in the control group. As illustrated in Supplementary Figure S2, the between-group differences in dimensional changes were clinically meaningful, particularly for intercanine width and arch perimeter, highlighting the enhanced transverse development associated with early myofunctional training.

### 3.4 Regression analysis

Multiple linear regression analysis was performed to identify factors associated with change in intercanine width as the primary outcome. The model included age, gender, type of habit, duration of habit, baseline intercanine width, and group allocation as independent variables. The overall model was significant (*F* = 8.42, *p* < 0.001, *R*^2^ = 0.458). Group allocation (intervention vs. control) emerged as the strongest predictor of intercanine width change (*β* = 0.52, *p* < 0.001), followed by younger age (*β* = −0.24, *p* = 0.028). Duration of habit and baseline measurements were not significant predictors in the adjusted model.

### 3.5 Adverse events

No serious adverse events were reported during the study. Minor issues included temporary gingival irritation from impression procedures in 4 children (2 in each group) and mild muscle fatigue following exercises in 6 children in the intervention group, which resolved without intervention. No participants discontinued due to adverse effects.

## 4 Discussion

The current controlled pilot study supports the fact that the early myofunctional training intervention, supplemented with habit cessation counseling, has a positive impact on maxillary arch development in children with mild cases of oral habits (4–7 years of age). The children who had the intervention therapy of 12 months myofunctional showed much higher increases in intercanine width, intermolar width and arch perimeter than the children that had the counseling intervention of habit cessation. There was also positive effect of the intervention group in palatal depth and increased rates of cessation of habits and improvement of the orofacial muscle functioning.

The observed enhancements in maxillary arch dimensions and palatal morphology can be mechanistically interpreted through the functional matrix hypothesis proposed by Moss, which posits that craniofacial skeletal growth is largely driven by the functional demands and activity of surrounding soft tissues rather than intrinsic genetic programming alone. In the present study, structured myofunctional training improved perioral muscle tone, lip seal competence, and tongue posture, thereby restoring a more physiological equilibrium of forces acting on the developing maxilla. Proper tongue elevation and resting against the palate generates intermittent light lateral and superior forces that stimulate the midpalatal suture. These forces promote sutural remodeling through mechanotransduction pathways, including activation of osteoblasts and osteoclasts, leading to appositional bone deposition along the suture margins and transverse expansion of the maxillary arch. Concurrently, enhanced lip seal and nasal breathing reduce aberrant buccal muscle pressures and open-mouth posture, which otherwise constrain lateral growth. The resultant transverse gains (evidenced by greater intercanine and intermolar width increases) and flattening of the palatal vault (reduced palatal depth) in the intervention group align with this model, as normalized orofacial muscle function appears to have amplified natural developmental stimuli during a period of high skeletal plasticity (prior to midpalatal suture maturation). This functional rebalancing likely mediated the superior arch perimeter expansion and habit cessation rates observed, supporting the premise that early correction of muscle dysfunction can positively modulate epigenetic influences on maxillary development.

The extent of the changes observed on dimensions of transverse arches in the intervention group is clinically significant. The average growth of 3.42 mm in intercanine width and 2.76 in intermolar width in 12 months is greater than the anticipated growth due to normal development in this developmental stage (22). The normative studies have also found that there are yearly increases in maxillary intercanine width of about 0.5–1.0 mm in the children aged 4–8 years which indicates that the changes in our intervention group are growth facilitating in addition to normal developmental progression (23).

Because the participants were aged 4–7 years, a period characterized by active craniofacial growth and dentition transition, part of the observed dimensional changes may reflect normal developmental processes rather than intervention effects alone. To address this issue, the present study included a parallel control group with comparable baseline characteristics and age distribution. Both groups demonstrated increases in arch dimensions over time, consistent with expected physiological growth; however, the magnitude of change was significantly greater in the intervention group. These between-group comparisons help distinguish treatment-related effects from age-related development. Furthermore, age was included as a covariate in regression analysis, where younger age showed a modest association with intercanine width change, while group allocation remained the strongest predictor. Nevertheless, some residual confounding from growth and tooth eruption cannot be completely excluded. In particular, intermolar width may be influenced by eruption of primary or permanent molars, and palatal depth may change as part of normal craniofacial maturation. Although the control group partially accounts for these developmental effects, the absence of skeletal maturity indicators or longitudinal normative growth references limits precise separation of treatment effects from natural growth. Future randomized studies incorporating growth-matched cohorts, skeletal maturation assessment, and longer follow-up periods are warranted to better isolate intervention-specific effects.

These findings can be explained by the functional matrix theory. Based on this concept, functional demands of skeletal structures affect their growth owing to the existence of soft tissue capsules around them (24). The tongue can influence the development of transverse by exerting physiological forces on the palate by proper establishment of tongue posture and function using myofunctional exercises. On the other hand, the faulty placement of the tongue, in connection with mouth behaviors, will not supply this stimulus, or will be constraining of the arch (25).

Of special interest is our results on the changes in the palatal depth. Reduced palatal depth in the intervention group with transverse expansion is indicative of the normalization of palatal morphology. There is also a tendency towards flattening of palates, which is usually related to mouth breathing and less contact of the tongue with palate, and observations of flattening palates are often attributed to a better relationship between the tongue and the palate (26). Other morphological changes observed in similar palatal in other studies including myofunctional therapy after myofunctional therapy in children with sleep-disordered breathing (27).

The increased rate of cessation of habits among those receiving the intervention (87.5% vs. 65.6) indicates that myofunctional training could help mitigate underlying issues that may be behind the inability of the target population to quit the habit. Oral habits are usually compensatory responses to orofacial muscle dysfunction or are habitual responses that are become a part of abnormal muscle responses (28). It can be suggested that by retraining proper muscle functioning, children can be less inclined to continue honing habits that had previously been performing a functional role. This fact is congruent with the earlier studies that showed that by treating the muscle dysfunction, the elimination of the habit may occur (29).

The fact that the scores of orofacial muscle functioning in the intervention group have advanced significantly justifies the effectiveness of the training program in terms of meeting its proximal objectives. The embellishments in muscle activity are likely to mediate the detected transformations in arch development, which together with the mechanistic pathway predicted by functional theories of craniofacial growth, uphold the mechanistic pathway (30). The fact that the better muscle function correlated with the dimensional changes in the arches, although it is not statistically significant in our regression model due to the sample size is something that should be further investigated.

When juxtaposing our study with the existing literature, it is important to note a number of areas of consistency and new contributions afforded by our study. Fellus, etc. found that myofunctional therapy had a positive effect on the development of the maxilla in children who had malocclusion, but their study did not include a control group, and they covered older children (31). Our findings were complemented by Cozza et al. who showed that early management of the oral habits using functional appliances could have a positive impact on development of the dental arch and this is the area that was combined with a behavioral intervention (32). This work stands apart as a significant portion of the extant literature is focused on secondary prevention.

The age group used in this study (47 years old) is a decisive intervention period. At this stage, the mid palatal suture is patent and is to functional pressure, there is growth of maxillary arch and children are developmentally fit to be put through organized exercise programs under parental guidance (33). Earlier intervention may have compliance problems whereas later intervention may have decreased skeletal responsiveness. The fact that younger age was linked with higher treatment response underpins the relevance of the early identification and treatment (34).

This study has a number of strengths that are worth noting. Compared to uncontrolled before-after studies, the controlled design that uses parallel groups and assigns them to various groups with different interventions makes it possible to make stronger causal assumptions. The 12 months follow-up duration is adequate to detect significant developmental changes and at the same time practical to a pilot study. Digital measures on 3D scans are accurate and reproducible, and assessor blinding can reduce assessor bias.

Nevertheless, there are significant weaknesses that should be taken into consideration. Although the quasi-randomized allocation procedure is feasible, it can be associated with selection bias in spite of the comparability of baseline. The nature of the intervention did not allow full blinding, which is likely to have affected parent-reported outcomes. Although the sample size is sufficient to identify the effects observed, subgroup analysis and evaluation of moderating factors are restricted. Also, the research was carried out in one centre and following a set of specific protocols, which could have an impact on future generalizability.

Although several outcomes reached statistical significance, their clinical relevance should also be considered. The observed increase in intercanine width (1.55 mm) may appear modest; however, even small transverse dimensional changes in children aged 4–7 years can be clinically meaningful. In early mixed dentition, a transverse gain of approximately 1–2 mm may contribute to increased arch perimeter, reduction in anterior crowding risk, and improved alignment of erupting permanent incisors. Moreover, early transverse development may help guide more favorable maxillary growth and reduce the need for more complex orthodontic intervention later. Therefore, the magnitude of change observed in the present study, although numerically small, is likely to be clinically relevant within the context of early interceptive treatment. Nevertheless, the clinical impact should be interpreted cautiously, and longer follow-up is required to confirm whether these dimensional improvements are maintained and translate into meaningful occlusal benefits.

The stability of the observed changes in the long term cannot be known and needs a long-term follow-up. The question of whether the gains in the development of the arches will be continued and whether they will lead to the lessening of the requirements of orthodontic treatment in adolescence is a crucial clinical issue that can be specified in future studies (35). The analysis of cost-effectiveness would also be useful in informing the healthcare policy choices about the preventive myofunctional programs.

Compliance in this research was quite high and close to 70% of the intervention subjects proved to be good adherents to the exercise program. This implies that myofunctional programs structured can be implemented in this age group using age-specific materials and parental guidance. Nevertheless, measures to enhance adherence in the subgroup of non-adhering families are to be created, which might involve electronic surveillance devices or gamification techniques.

Clinically, the findings may justify the incorporation of myofunctional assessment and training into the pediatric dental practice, especially in cases where children are already exhibiting oral habitual practices. Proactive intervention and early screening of orofacial muscle dysfunction could be a beneficial preventive orthodontic measure. Pediatric dentists, orthodontists, and myofunctional therapists should collaborate in order to optimize the detection and treatment of at-risk children.

The quasi-randomized allocation based on recruitment week represents another limitation. Although a predefined schedule was used and baseline characteristics were comparable, the absence of true random sequence generation and allocation concealment may introduce selection bias and reduce internal validity. Future studies should employ fully randomized allocation with concealed sequence generation to confirm these findings.

Some secondary outcomes, including habit frequency and compliance, were based largely on parental reporting, which may be subject to reporting and recall bias. Although structured recording forms and investigator review were used to improve accuracy, objective compliance monitoring was not available. Therefore, the reported adherence levels should be interpreted cautiously, and future studies incorporating objective compliance tracking methods are recommended to validate these findings.

Further studies need to involve a bigger number of multicenter randomized controlled trials with long-term follow-up to validate these initial results. The exploration of the best exercise regimens, treatment length, and maintenance procedures would aid in the improvement of the clinical guidelines. Relative research comparing myofunctional therapy with habit counseling and combined therapy would help understand the relative roles of each of the components. Last, a study that depicts the neurophysiological and biomechanical processes of how the theoretical effects are realized would contribute to the theoretical knowledge. Overall, while the present findings are encouraging, they remain preliminary and should be viewed in the context of the pilot nature of this single-center study.

## 5 Conclusion

This controlled pilot study provides preliminary evidence that early myofunctional training, when combined with habit cessation counseling, may positively influence maxillary arch development in children aged 4–7 years with mild oral habits. Over the 12-month intervention period, children who received structured myofunctional exercises demonstrated greater increases in maxillary intercanine width, intermolar width, and arch perimeter compared with those receiving standard habit cessation counseling alone. The intervention group also showed favorable changes in palatal depth, higher rates of complete habit cessation, and significant improvements in orofacial muscle function. These findings suggest that structured myofunctional exercise programs may support transverse maxillary development and facilitate habit elimination in young children during a period of high craniofacial plasticity. Clinically, the results indicate that early identification of children with oral habits and orofacial muscle dysfunction, followed by myofunctional therapy, could represent a promising preventive orthodontic approach. Such an intervention may help reduce the severity of emerging malocclusion and the potential need for more extensive orthodontic treatment later. However, as this was a single-center pilot study with quasi-randomized allocation and a relatively small sample, the findings should be interpreted cautiously. Larger multicenter randomized controlled trials with longer follow-up are warranted to confirm these preliminary observations, assess long-term stability of the arch dimensional changes, and evaluate the clinical impact on occlusal outcomes.

## Data Availability

The datasets presented in this study can be found in online repositories. The names of the repository/repositories and accession number(s) can be found in the article/Supplementary Material.
