# Supplementary material for: Influence of early myofunctional training on maxillary arch development in children aged 4–7 years with mild oral habits: a controlled pilot study
Source: Front Pediatr. 2026 Jul 7;14:1809806. doi: 10.3389/fped.2026.1809806 (PMC13384936; doi:10.3389/fped.2026.1809806)
Supplement: Supplementary file 1 [file Supplementaryfile1.docx]

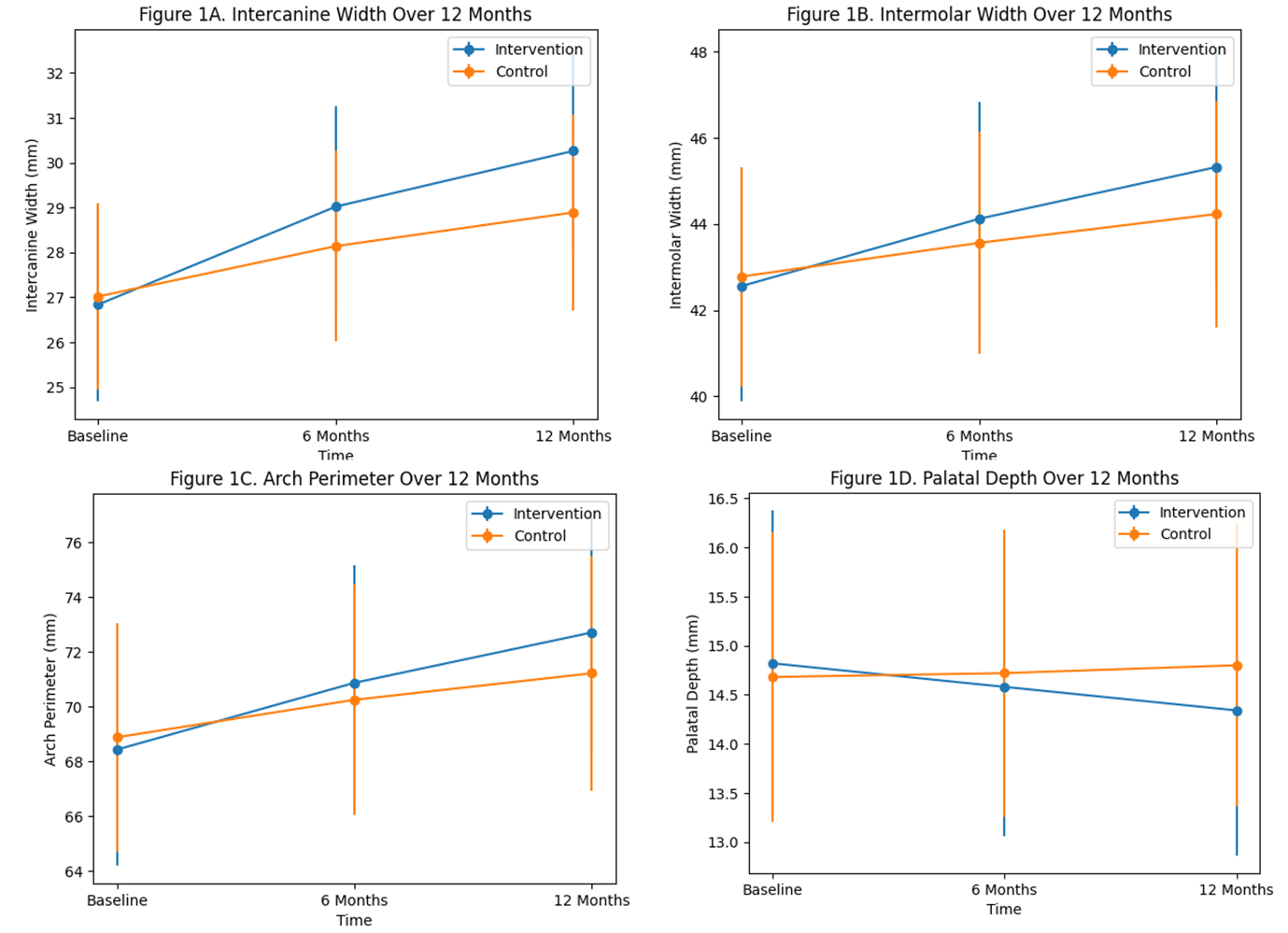


**Figure S1.** Longitudinal changes in maxillary arch dimensions over 12 months. Mean values (±SD) of (A) intercanine width, (B) intermolar width, (C) arch perimeter, and (D) palatal depth are shown for intervention and control groups at baseline, 6 months, and 12 months. The intervention group demonstrated significantly greater transverse expansion and arch perimeter increase, along with reduced palatal depth compared to controls (p < 0.001).


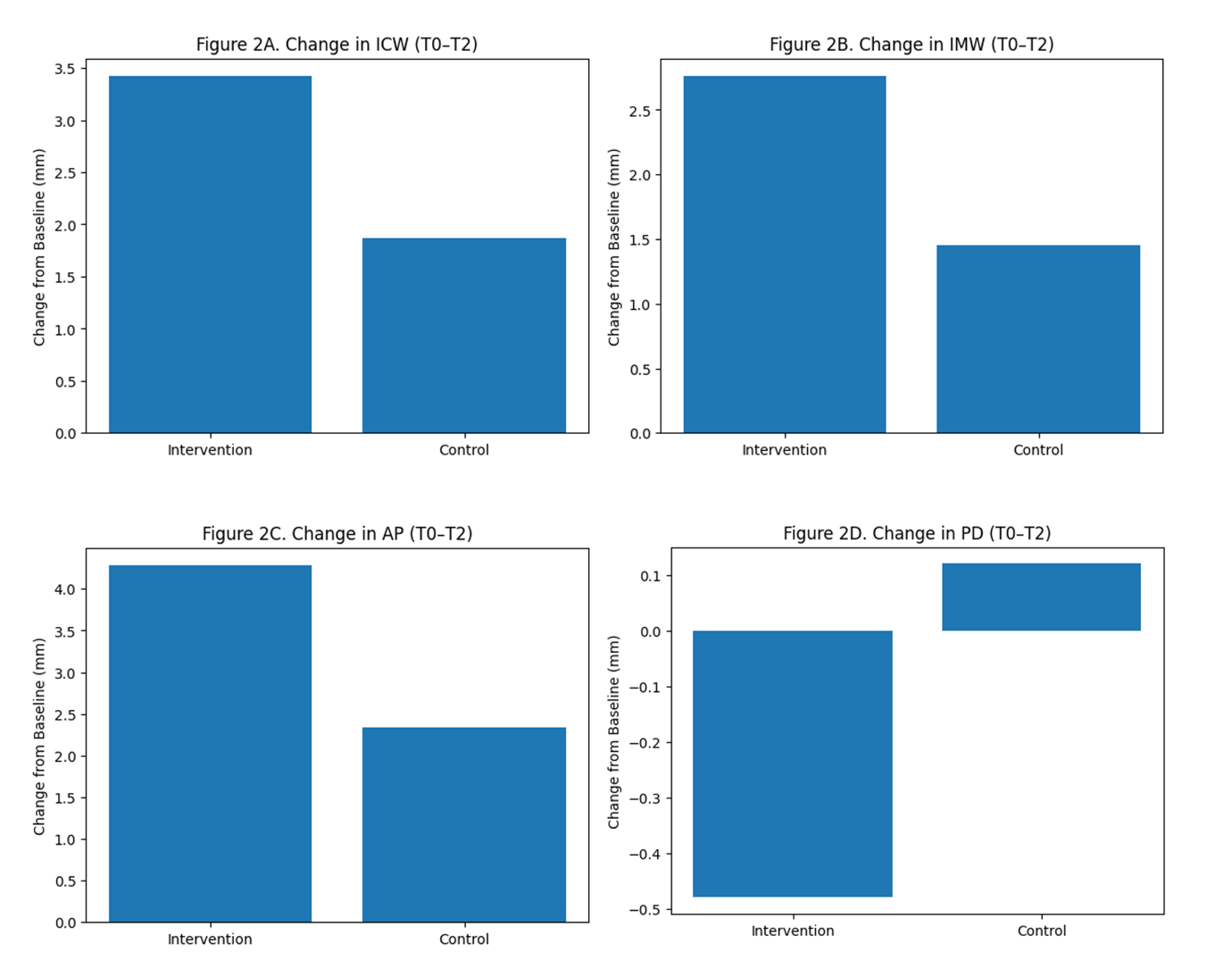


### **Figure S2. Mean Changes in Maxillary Arch Dimensions From Baseline to 12 Months (T0–T2).** Bar graphs illustrating the mean change (mm) in (A) intercanine width (ICW), (B) intermolar width (IMW), (C) arch perimeter (AP), and (D) palatal depth (PD) after 12 months in the intervention and control groups. The intervention group demonstrated significantly greater increases in ICW, IMW, and AP compared with the control group (p < 0.001 for all). Palatal depth decreased in the intervention group, indicating reduced palatal vault height, whereas minimal change was observed in controls (p < 0.001). Positive values represent dimensional increases; negative values represent decreases.
